# Allicin protects against myocardial I/R by accelerating angiogenesis via the miR-19a-3p/PI3K/AKT axis

**DOI:** 10.18632/aging.203578

**Published:** 2021-10-04

**Authors:** Mengru Liu, Peng Yang, Dongliang Fu, Tong Gao, Xinyi Deng, Mingjing Shao, Jiangquan Liao, Hong Jiang, Xianlun Li

**Affiliations:** 1 Graduate School of Peking Union Medical College, Beijing 100730, China; 2 Department of Integrative Medicine Cardiology, China-Japan Friendship Hospital, Beijing 100029, China; 3 Peking University China-Japan Friendship School of Clinical Medicine, Beijing 100029, China

**Keywords:** myocardial I/R, miR-19a-3p, PI3K/AKT pathway, angiogenesis, Allicin

## Abstract

Objectives: Allicin is an allyl 2-propenethiosulfinate or diallyl thiosulfinate acid with cardioprotective effects in myocardial ischemia/reperfusion (MI/R) injury. This study aims to examine the underlying mechanism by which Allicin protects against MI/R.

Methods: C57BL6 mice were subjected to either sham or MI/R surgery, and mice in the Allicin group were injected with Allicin (5 mg/ml) before the induction of ischemia. The cardiac function and histopathology of experimental mice were evaluated by ultrasound quantification and Masson staining. We next measured the capillary angiogenesis of the peri-infarct area by Masson staining and immunohistochemical staining. The miRNA microarray was carried out to examine the expressed miRNAs in MI/R tissues and corresponding normal tissues. Real-time quantitative polymerase chain reaction (q-PCR) was performed to validate the selected miRNA-19α-3p gene expression. Besides, we evaluated the myocardial lactate dehydrogenase and COX-2 by immunofluorescence staining. The western blot analysis was used to evaluate the protein levels of p-AKT, p-PI3K, p-mTOR, COX-2, and VEGF protein in the Allicin and Model group. *In vitro* study, LPS stimulated Tie2 expressing macrophages were cultured in an ischemic buffer. We evaluated the accumulation of VEGF by fura-2/AM fluorescence. Besides, Western blotting was performed to examine the protein levels of p-PI3K, p-AKT, p-mTOR, VEGF, COX2, and MMP2. The PI3K inhibitor was applied to investigate whether Allicin-induced myocardial ischemia-reperfusion injury protection is mediated via the PI3K/AKT pathway. And the miR-19α-3p mimic/inhibitor were transfected to promote/inhibit the expression of miR-19a-3p for verifying the regulation of miR-19a-3p on PI3K pathway.

Results: Allicin pretreatment significantly improved I/R-induced cardiac function damage. Furthermore, Allicin could repress cardiac fibrosis, as evidenced by reduced areas of cardiac fibrosis. Allicin’s effect on the MI/R was associated with increased capillary angiogenesis. Microarray analysis exposed that miR-19a-3p down-regulated PIK3CA (PI3K) expression by directly targeting the PIK3CA gene. The regulation of the angiogenesis pathway and gene miRNA-19a-3p might affect the Allicin-induced MI/R protection. Immunofluorescence staining revealed that COX-2 and myocardial lactate dehydrogenase were significantly increased after Allicin treatment. Furthermore, western blot analysis demonstrated that p-AKT, p-PI3K, p-mTOR, COX-2, and VEGF protein levels were also increased in the Allicin group. *In vitro* study, the protein levels of p-PI3K, p-AKT, p-mTOR, VEGF, COX2, and MMP2 were significantly increased in the Allicin-treated Tie2 expressing macrophages. These effects were partially reversed by PI3K inhibitor (Wortmannin) treatment. MiR-19α-3p plays an important role in myocardial I/R injury. It could regulate the activity of the PI3K-AKT pathway. And inhibition of miR-19a-3p promoted angiogenesis by regulating PI3K/AKT pathway.

Conclusions: Allicin pretreatment protects against myocardial I/R and activating the miR-19a-3p/PI3K/AKT pathway.

## INTRODUCTION

Ischemic heart disease (IHD) is one of the leading causes of death and disability globally [1, 2]. Although there are many novel therapeutic interventions available for myocardial ischemia treatment, the mortality and morbidity rate are increasing [3]. Reinstating blood flow supply to ischemic myocardial could improve myocardial viability, reduce myocardial infarct size [4]. Notwithstanding, reinstating blood supply is often followed by reperfusion arrhythmia and myocardial stunning, known as myocardial ischemia-reperfusion (I/R) injury, which makes myocardial damage more serious by accelerating myocardial death, cardiac dysfunction, and ventricular remodeling [5]. Severe myocardial ischemia-reperfusion (I/R) injury can induce myocardial necrosis, cardiac dysfunction, myocardial infarction, and heart failure (HF) [6, 7]. Currently, there is no effective solution to avoid MI/R. Consequently, the search for novel therapeutic strategies for MI/R is essential. In this context, we used an Allicin-based approach to investigate the underlying mechanism of MI/R injury to explore novel intervention strategies.

Allicin (2-Propene-1-sulfinothioic acid S-2-propenyl ester, diallyl thiosulfinate), a reactive sulfur species with several diverse biological properties, is responsible for the unique smell and taste of garlic [8]. Accumulated biochemical data have shown that Allicin could improve physical health. It can improve many cardiovascular diseases, including arteriosclerosis, cerebral infarction, arrhythmia, and hypertension [9, 10]. Phosphatidylinositol-3 kinases (PI3K), a heterodimeric lipid kinases family, are responsible for coordinating a diverse range of cellular functions [11]. Additionally, various studies have confirmed that PI3K/AKT pathway plays a crucial role in regulating various cellular functions, including metabolism, proliferation, survival, and transcription [12, 13]. Remarkably, it has been shown that microRNAs are a kind of small non-coding RNA that contain 20–22 nucleotides, which participate in multiple physiological and pathophysiological processes such as apoptosis, proliferation, and differentiation [13]. The generally conserved miR-19a-3p has recently attracted our attention, which could regulate cell proliferation, apoptosis, and inflammatory responses [14, 15]. Therefore, the present study aimed to investigate the use of Allicin in MI/R and to elucidate whether the effects of Allicin are mediated by the miR-19a-3p and PI3K/AKT pathways.

## METHODS

### Animals

Adult male C57BL6 mice (purchased from the experimental animal center, Henan, China) were maintained under a 12-hour light/dark cycle at 25°C air-conditioned with unrestricted access to standard food and water. To determine if the PI3K is involved in Allicin-induced MI/R protection, mice were randomly divided into three groups (*n* = 20 per group): 1) Sham group; 2) Myocardial I/R group (Model); 3) Allicin + MI/R group (Allicin). The Allicin group mice were then treated with Allicin (3 mg/ml × 10 min + 5 mg/ml × 15 min, intravenously) before undergoing myocardial I/R. The mice in the sham group and the MI/R group were treated with the same volume of saline before undergoing myocardial I/R.

### Myocardial I/R mice model

Mice were anesthetized with 1% pentobarbital sodium (50 mg/kg, Sigma, St. Louis, USA) by intraperitoneal injection. Tracheal intubation and mechanical ventilation were conducted with a rodent ventilator (ALC-V8, Shanghai, China). The skin of the chest was disinfected, and a left thoracotomy was performed at the fourth and fifth intercostal spaces to uncover the heart. The left anterior descending artery (LAD) of the coronary artery was ligated using an 8–0 silk thread for 30 minutes of ischemia treatment and then released to achieve reperfusion for 180 min. The ischemia criteria included light colored myocardium and the color changes in the ischemic area. The reperfusion was achieved via releasing the knot and sutured the incision. The mice in the sham group were operated in the same way but without ligating the LAD branch.

### Cell culture and simulated Myocardial I/R *in vitro* model

Tie2 expressing macrophages were collected form I/R mice by flow cytometry sorting and then the slides of cells were photographed after immunofluorescent staining for Tie2. To simulate ischemia/reperfusion injury *in vitro*, Tie2^+^ macrophages were cultured in an ischemic buffer for 1h in a cell culture incubator as previously described [16, 17]. Then, serum-free DMEM containing Reperfusion was conducted by exposing the cells to serum-free DMEM with or without Allicin for 4 h. Cells were washed and stimulated with LPS (10 ng/ml) and incubated at 37°C for 24 h. Cells were then treated with Allicin and saline for an additional 4 h, then consigned in an ischemic buffer for an additional1h followed by incubation in serum-free DMEM for 4 h. The Tie2^+^ macrophages were transfected miR-19a-3p NC, mimic, inhibitor by lipo 3000 and then the protein was collected and carried on the western blot experiments.

### Echocardiography

After MI/R, mice were subjected to echocardiography using a high-resolution ultrasound imaging system (Vevo 2100, VisualSonics) for cardiac function measurement. Left ventricular ejection fraction (LVEF) and fractional shortening (LVFS) were calculated. The left ventricle inner diameter during diastole (LVIDd) and left ventricle anterior wall thickness during diastole (LVAWd) were measured. LVPW;d and LVAWs were calculated.

### Masson staining and immunostaining

Paraffin-embedded cross-sections of the mice myocardial specimens (4-μm thick) were stained with Masson stain (Sigma-Aldrich, St. Louis, MO, USA) according to standard protocols [18]. For immunofluorescence staining, paraffin-embedded cross-sections were deparaffinized, rehydrated, and then subjected to antigen retrieval. Cells were washed three times in 1 × PBS in fluorescence medium at 4°C. Cells and tissue sections were incubated with primary antibody (Abcam, 1:50 dilution) at 4°C overnight, followed by incubation with secondary antibodies (Abcam, 1:150 dilution) for 2–3 h at 37°C. Cells were then splashed in 1 × PBS. Fluorescence was viewed with Olympus DP-50 fluorescent microscope.

### Real-Time qPCR

Total RNA was extracted using Trizol reagent, and quantitative polymerase chain reaction (qPCR) package compels a real-time PCR machine (Bio-Rad, USA). The standard curve was attained cycle threshold (Ct) by qPCR reaction cycle curve. Fold change = 2^−ΔΔCt^, and data were analyzed with StepOne software (Applied Biosystems, CA).

### Western blot analysis

At the end of the reperfusion, cardiac tissues were harvested and lysed with cold radioimmunoprecipitation assay (RIPA) buffer (Beyotime Institute of Biotechnology), supplemented with protease inhibitor cocktail (1:100, Sigma-Aldrich). Total protein was extracted from the MI area. After protein concentration was determined using the Bradford assay (Bio-Rad Laboratories, Hercules, CA, USA), the equal protein samples were separated in 10% SDS-polyacrylamide gel electrophoresis and transferred onto a nitrocellulose membrane. Then, they were incubated with primary antibodies against PI3K, p-AKT, AKT, p-PI3K, p-mTOR, VEGF, COX2, mTOR, MMP2, and β-actin (1: 1000, Cell Signaling Technology, MA, USA) at 4°C overnight, and followed by incubation with corresponding secondary antibodies (1: 5000, R&D, Minneapolis, MN, USA) at RT for 1 h. The immunoblots were identified using an image analyzer Quantity One System (Bio-Rad, CA, USA). The results were normalized to the β-actin protein levels.

### Statistical analyses

Data in the current study were exhibited as mean ± SEM. Differences between the two groups were estimated with Student’s *t*-test. One-way ANOVA estimated differences between more than two groups. *P* < 0.05 was considered significant. SPSS 20.0 software was used for statistical analysis.

### Data availability

The data used to support the findings of this study are included in the article.

### Ethics approval

The animal use protocol for this study has been reviewed and approved by the Animal Care Welfare Committee, China-Japan Friendship Hospital.

## RESULTS

### Allicin attenuated myocardial ischemia-induced cardiac fibrosis and improved cardiac function

**Figure 1:**
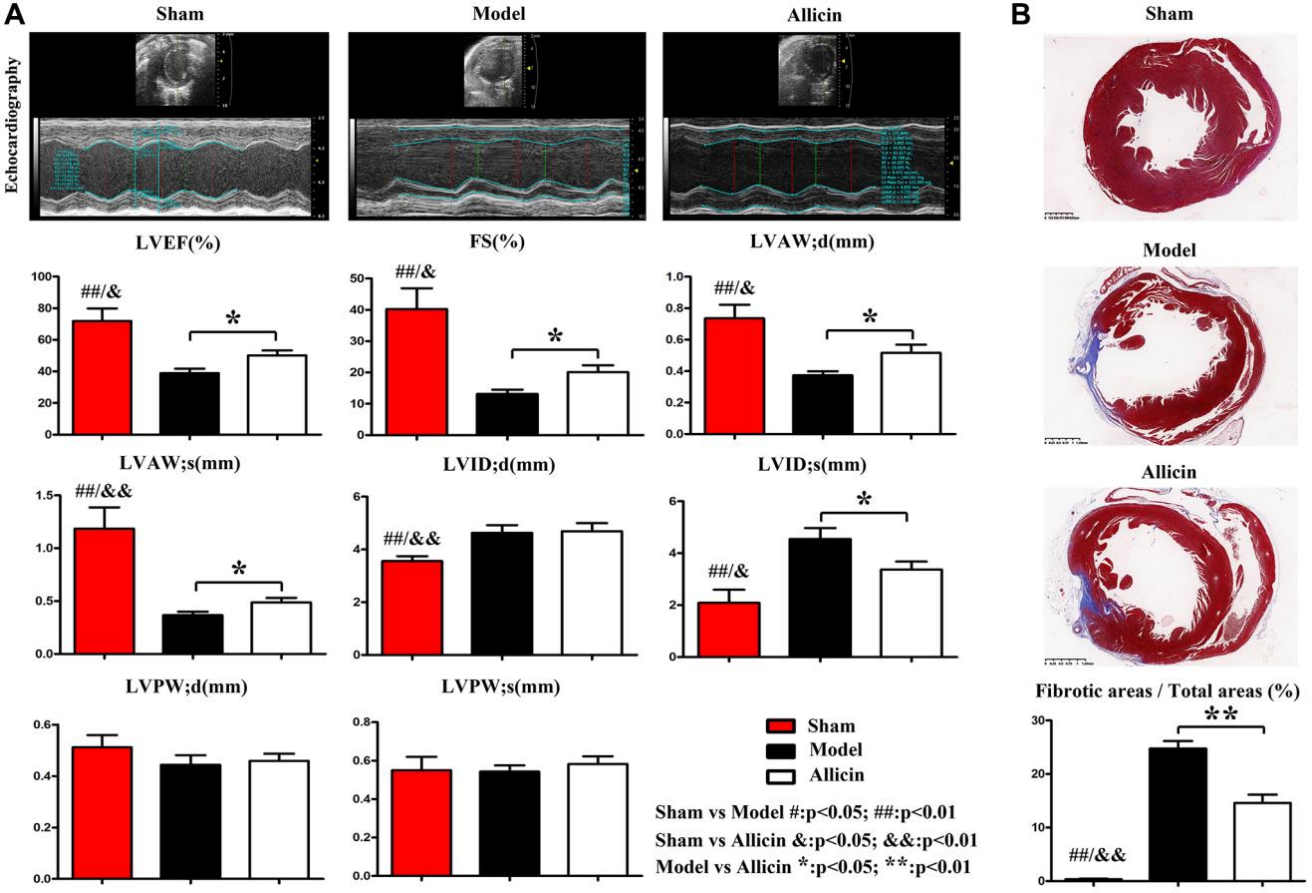
**Allicin attenuated myocardial ischemia-induced cardiac fibrosis and improved cardiac function.** (**A**) Representative images of the transabdominal ultrasound imaging were presented. Compared with the Control group, the values of LVEF, LVFS, LVAW;d and LVAW;s were significantly decreased in Model group, while LVIDd and LVIDs were increased (^*^P < 0.01). Compared with the Model group, the LVEF, LVFS, LVAW;d and LVAW;s were increased in the Allicin group, whereas LVID;s was decreased (^*^P < 0.05). (**B**) Masson staining demonstrated that the areas of cardiac fibrosis of the Model group mice were significantly increased compared with the Control group mice's hearts. And the areas of cardiac fibrosis of the Model group mice were significantly increased compared with Allicin group mice (^*^P < 0.05).

The cardiac function of each group was evaluated by ultrasound quantification. Representative images of the transabdominal ultrasound imaging from the Control, Model and Allicin group were presented in Figure 1A. Compared with the Control group, the values of LVEF, LVFS, LVAW;d and LVAW;s were significantly decreased in Model group (^*^P < 0.01), while LVIDd (^*^P < 0.01) and LVIDs (^*^P < 0.01) were increased. Echocardiography of the Allicin group exhibited a noticeable increase in the LVEF, LVFS, LVAW;d and LVAW;s compared with the Model group (^*^P < 0.05). Additionally, LVID;s of the Allicin group was significantly decreased compared with the Model group (^*^P < 0.05). The histopathology of experimental mice from the Model and Allicin group was evaluated by Masson staining. Masson staining analysis demonstrated that the areas of cardiac fibrosis of the Model group mice were significantly increased compared with the Control group mice’s hearts. Allicin could repress cardiac fibrosis, as evidenced by reduced cardiac fibrosis areas in the Allicin group mice compared with the Model group (Figure 1B, ^*^P < 0.05). These results implied that cardiac function was decreased and cardiac remodeling occurred in mice with myocardial ischemia reperfusion. However, Allicin attenuated myocardial ischemia-induced cardiac fibrosis and improved cardiac function.

### Allicin’s effect on the MI/R was associated with increased capillary angiogenesis in the peri-infarct area

**Figure 2:**
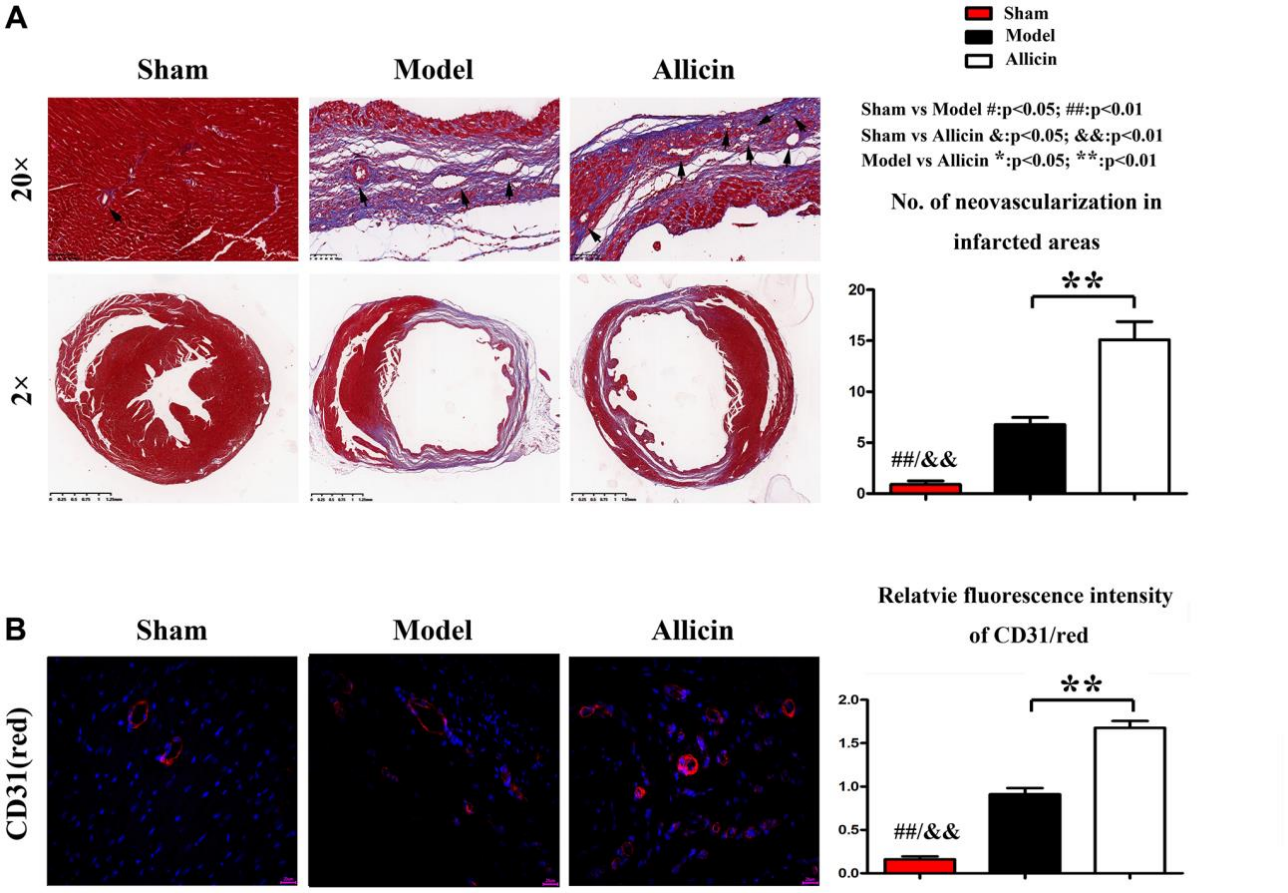
**Allicin’s effect on the MI/R was associated with increased capillary angiogenesis in the peri-infarct area.** (**A**) Masson staining of myocardial sections showed that the numbers of neovascularization was increased in myocardium Model group compared with the Control group, and the Allicin group's capillary angiogenesis was increased compared with the Model group (^*^P < 0.01). (**B**) Immunohistochemical staining for myocardial lactate dehydrogenase (green) and CD31(red) in the peri-infarct area showed that Compared with the Control group, the expression level of CD-31 was upregulated in myocardium Model group, while Allicin treatment further increased capillary angiogenesis compared with the Mode (^*^P < 0.01).

To determine the role of Allicin in capillary angiogenesis of the peri-infarct area, we performed Masson staining and immunohistochemical staining. Masson staining of myocardial sections showed that the numbers of neovascularization were increased in myocardium Model group compared with the Control group, and the Allicin group's capillary angiogenesis was increased compared with the Model group (Figure 2A). We further determined the relative numbers of neovascularization in infarcted areas. As shown in Figure 2A, the Allicin treatment significantly increased the neovascularization numbers compared with the Model group (^*^P < 0.01). Capillary angiogenesis was further examined by immunohistochemical staining for CD-31 in the peri-infarct area. Compared with the Control group, the expression level of CD-31 was upregulated in myocardium Model group, while Allicin treatment further increased capillary angiogenesis compared with the Model (Figure 2B). Quantification indicated that the relative fluorescence intensity of CD-31 was significantly higher in the Allicin group than the Model group (^*^P < 0.01). These findings suggested that mice in Model group had initiated the angiogenesis mechanism compared with the Control group, but it was not enough. Allicin could further promote angiogenesis, thereby improving MI/R injury. Allicin’s effect on the MI/R was associated with increased capillary angiogenesis in the peri-infarct area.

### Allicin increased Cox-2, VEGF, myocardial LDH, and activated PI3K/AKT/mTOR pathway

**Figure 3:**
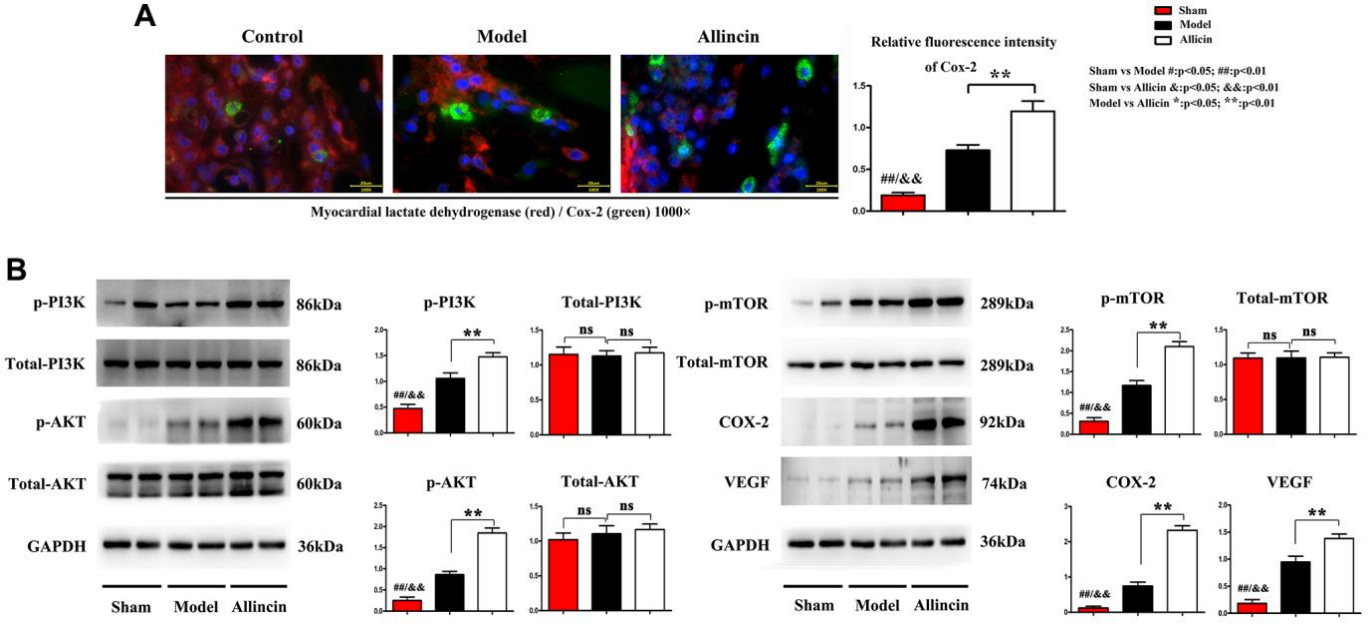
**Allicin increased Cox-2, VEGF, myocardial LDH, and activated PI3K/AKT/mTOR pathway.** (**A**) The immunofluorescence staining showed that compared with the Control group, the COX-2 positive area in the myocardial infarction area of Model group increased. Compared with the Model group, the myocardial LDH and COX-2 positive area was further significantly increased after Allicin treatment (^*^P < 0.01). (**B**) Western blot analysis demonstrated that p-AKT, p-PI3K, p-mTOR, COX-2, and VEGF protein levels were also increased in the Allicin group compared to the Model (^*^P < 0.01).

Published studies indicate that cyclooxygenase-2 (COX-2) is the inducible enzyme involved in prostaglandin synthesis, and COX-2 plays a vital role in neovascularization by regulating vascular endothelial growth factor (VEGF) and fibroblast growth factor, migration of endothelial cells. Lactate dehydrogenase (LDH) is the primary metabolic enzyme that plays a significant role in regulating myocardial bioenergetics [19, 20]. The immunofluorescence staining of COX-2(green) and myocardial lactate dehydrogenase (red) in the peri-infarct area was performed. Compared with the Control group, the COX-2 positive area in the myocardial infarction area of Model group increased. Compared with the Model group, the myocardial LDH and COX-2 positive area was further significantly increased after Allicin treatment. Quantification indicated that the relative fluorescence intensity of Cox-2 was significantly higher in the Allicin group compared with the Model group (Figure 3A, ^*^P < 0.01).

Furthermore, western blot analysis demonstrated that p-AKT, p-PI3K, p-mTOR, COX-2, and VEGF protein levels were also increased in the Allicin group compared to the Model. These findings were further supported by qPCR analysis of protein levels of p-AKT, p-PI3K, p-mTOR, COX-2, and VEGF (Figure 3B, ^*^P < 0.01). These data supported the conclusion of microarray analysis that the PI3K/Akt/mTOR pathway was involved in the suppressive effect of Allicin in the development of MI/R.

### Microarray analysis exposed the regulation of the angiogenesis pathway, and gene miRNA-19a-3p might affect the Allicin-induced MI/R protection

**Figure 4:**
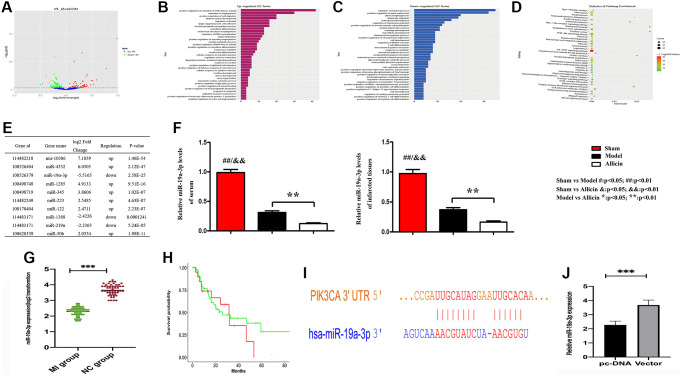
**Microarray analysis exposed the regulation of angiogenesis pathway, and gene miRNA-19a-3p might affect the Allicin-induced MI/R protection.** (**A**) The volcano map showed the differently expressed miRNAs by the criteria of |fold change| >2 and *P* < 0.05 between MI/R and normal controls. (**B**–**C**) Functional classification of these differentially expressed genes based on the Gene Ontology (GO). (**D**) Partial results of the KEGG pathways. (**E**) The top 10 differentially expressed miRNAs in myocardium Model group compared with the Control group. (**F**) RT-PCR indicate that MI/R could induce the expression of miR-19a-3p, which can be further modulated by Allicin. (**G**) RT-PCR revealed that miR-19a-3p has downregulated in mice MI/R specimens compared with matched control tissues. (**H**) The Kaplan-Meier survival curve was performed to evaluate the diagnostic value of miR-19a- 3p in MI/R. (**I**) The potential binding site between miR-19a-3p and PIK3CA (PI3K) by Miranda. (**J**) The expression levels of miR-19a-3p were noticeably down-regulated in PI3K overexpressed macrophages.

The miRNA microarray was carried out to examine the expressed miRNAs in MI/R tissues and corresponding normal tissues. We identified differently expressed miRNAs by the criteria of |fold change| >2 and *P* < 0.05 between MI/R and normal controls, a well-defined distinction on the significantly different expression of miRNAs was depicted in the volcano map. 67 genes were significantly downregulated, and 68 genes were upregulated (Figure 4A). Functional classification of these differentially expressed genes based on the Gene Ontology (GO) annotations was performed and found 35 up-regulated pathways (Figure 4B), including regulation of angiogenesis and regulation of growth. Furthermore, we also found 43 down-regulated pathways (Figure 4C). Similarly, partial results of the KEGG pathways were shown in Figure 4D. Several previous studies highlight that the PI3K/ARK pathway plays a crucial role in the activation of the mTOR signaling pathway, promoting angiogenesis regulation [21]. We have found that Allicin protects against myocardial IR by accelerating angiogenesis via the PI3K/AKT axis. Therefore, we wanted to further explore the molecular mechanism of the protective role of Allicin and select miRNAs with statistical difference and related to angiogenesis for further study.

There are top 10 differentially expressed miRNAs in myocardium Model group compared with the Control group. Among them, we selected a miRNA for further investigation, miR-19a-3p, which served a critical role in cerebral ischemia/reperfusion (Figure 4E) [22]. Previous study indicated that miR-19a-3p inhibited endothelial cells proliferation and angiogenesis via targeting HIF-1α and attenuated heart function of mice after MI, and antagomiR-19a-3p treatment accelerated angiogenesis [23]. In our experimental results, neovascularization was seen in the myocardial infarction area of Model group, but not enough. And the expression of miR-19a-3p was significantly down-regulated in Model group. However, Allicin could further promote angiogenesis. We found that the expression of miR19a-3p in Allicin group was further reduced. Furthermore, we also collected the culture medium of serum and analyzed the level of miR-19a-3p, shown in Figure 4F. As a result, it also downregulated in response to the stimulation of HR, and it was further decreased in the Allicin group. These results indicate that MI/R could induce the expression of miR-19a- 3p, which can be further modulated by Allicin. Additionally, RT-PCR revealed that miR-19a-3p has downregulated in mice MI/R specimens compared with matched control tissues (Figure 4G). The Kaplan-Meier survival curve was performed to evaluate the diagnostic value of miR-19a-3p in MI/R. We found that miR-19a-3p might be a worthy diagnostic marker for MI/R (Figure 4H), suggesting that miR-19a-3p might play an essential role in MI/R development.

Earlier work indicated that miR-19a-3p could activate PTEN and inhibit the PI3K/AKT pathway [24]. Next, we investigated the relationship between miR-19a-3p and PI3K/AKT pathway, and we found the potential binding site between miR-19a-3p and PIK3CA (PI3K) by Miranda (Figure 4I). And the expression levels of miR-19a-3p were noticeably down-regulated in PI3K overexpressed macrophages (Figure 4J).

Therefore, combined with the function and the frequency of genes in enriched pathways associated with MI/R disease and our own experimental results, we finally chose miR-19a-3p and regulation of angiogenesis pathway for further investigation.

### Allicin-mediated increase in VEGF and MMP was dependent on pPI3K

**Figure 5:**
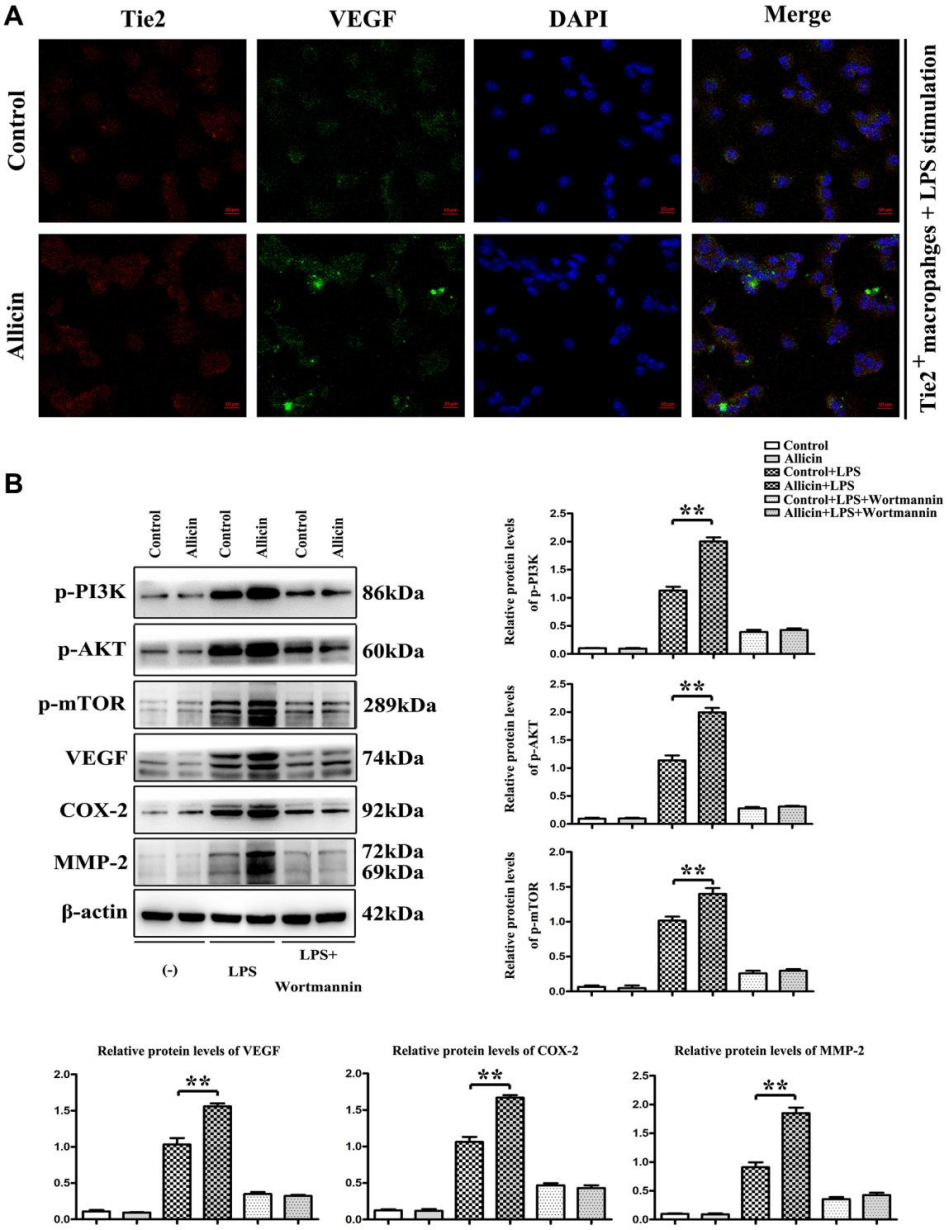
**Allicin-mediated increase in VEGF and MMP was dependent on pPI3K.** LPS stimulated Tie2 expressing macrophages were cultured in an ischemic buffer. Tie2 expressing macrophages were incubated with fluorescent dye fura-2/AM. (**A**) The accumulation of VEGF was noticeably visualized with fura-2/AM fluorescence in the Allicin treated Tie2 expressing macrophages. (**B**) the results of qPCR and Western blotting showed that Allicin treatment significantly increased the expression of the p-PI3K, p-AKT, p-mTOR, VEGF, COX2, and MMP2 in LPS stimulated Tie2 expressing macrophages compared with saline control (*P* < 0.05). Furthermore, we found the opposite results that the increased p-PI3K, p-AKT, p-mTOR, VEGF, COX2, and MMP2 were reversed by inhibitor treatment (*P* < 0.05).

To validate the protective effect of Allicin on MI/R *in vitro*, Tie2 expressing macrophages were treated with Allicin and saline control. Evidence has indicated that LPS is a well-known M1 inducer that could produce proinflammatory cytokines in macrophages [25]. Tie2 expressing macrophages /monocytes (TEMs) play essential roles in the neovascularization of ischemic tissues [26, 27]. In this present study, LPS stimulated Tie2 expressing macrophages were cultured in an ischemic buffer. Tie2 expressing macrophages were incubated with fluorescent dye fura-2/AM. The VEGF accumulation was noticeably visualized with fura-2/AM fluorescence in the Allicin-treated Tie2 expressing macrophages (Figure 5A). Besides, qPCR and Western blotting results showed that Allicin treatment significantly increased the expression of the p-PI3K, p-AKT, p-mTOR, VEGF, COX2, and MMP2 in LPS stimulated Tie2 expressing macrophages compared with saline control (Figure 5B, ^*^P < 0.05). Next, we determined if PI3K pathway activation regulates VEGF and MMPs expression in Tie2 expressing macrophages. A PI3K inhibitor named Wortmannin was injected into the culture medium of LPS stimulated TEMs from the control and Allicin group. As expected, we found the opposite results. The increased p-PI3K, p-AKT, p- mTOR, VEGF, COX2, and MMP2 were reversed by inhibitor treatment (Figure 5B). Taken together, these data supported the conclusion that the Allicin mediated increase in VEGF and MMP was dependent on pPI3K, and Allicin significantly attenuated the formation and severity of myocardial ischemia injury via miR-19a-3p/PI3K/AKT signaling pathway.

### MiR-19a-3p promotes angiogenesis by regulating PI3K/AKT pathway

**Figure 6:**
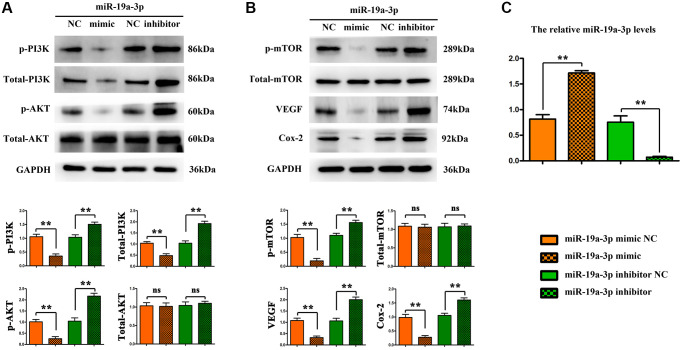
**MiR-19a-3p promotes angiogenesis by regulating PI3K/AKT pathway.** (**A**–**B**) Compared with the control group, the expression of p-PI3K, p-AKT, p-mTOR, VEGF, Cox-2, MMP-2 were significantly decreased after miR-19α-3p mimic transfection, and further significantly increased after miR-19α-3p inhibitor transfection. (^*^P < 0.01). (**C**) The miR-19a-3p levels of each groups.

Furthermore, we verify the regulation of miR-19a-3p on PI3K pathway by using neonatal mice tie2^+^ macrophages using the flow cytometry sorting method. MiR-19α-3p mimic/inhibitor were transfected to promote/inhibit the expression of miR-19a-3p, miRNA control and inhibitor control transfection being taken as control. And then, mice tie2^+^ macrophages were oxygenated for 4 hours and then reoxygenated for 2 hours to simulate the ischemia-reperfusion model *in vitro*. Compared with the control group, the expression of p-PI3K, p-AKT, p-mTOR, VEGF, Cox-2, MMP-2 were significantly decreased after miR-19α-3p mimic transfection, and further significantly increased after miR-19α-3p inhibitor transfection (Figure 6A and 6B). The miR-19α-3p levels of each groups were shown in Figure 6C. These data suggest that inhibition of miR-19a-3p promotes angiogenesis by regulating PI3K/AKT pathway.

## DISCUSSION

Myocardial ischemia-reperfusion (MI/R) injury has been one of the major fatal diseases in China [28]. Besides sudden death, MI/R also results in various clinical manifestations such as cardiac hypertrophy and coronary disease [29]. Allicin is a defensive molecule from garlic (Allium sativum) with a wide range of biological activities [8]. This study was designed to investigate the function of Allicin against MI/R injury and expose the underlying mechanisms. The results demonstrated that Allicin served a significant role in protecting against myocardial ischemia-reperfusion *in vivo* and *in vitro*. We first observed the role of Allicin served in myocardial I/R injury mice models, which were ligated for 30 min and reperfusion for 180 min. Through the heart hemodynamic function investigation, cardiac function was decreased and cardiac remodeling occurred in Model group, while Allicin treatment increased LVEF and LVFS, repressed cardiac fibrosis, and decreased the LVAD, LVAW;d, and LVAW;s in the mice. Besides, the Masson staining and immunohistochemical staining results revealed that angiogenesis increased but not enough after myocardial I/R, and the protective effect of Allicin on the MI/R was derived from increasing capillary angiogenesis in infarcted areas.

In recent years, an increasing number of studies have suggested that cyclooxygenase-2 (COX-2) is the inducible enzyme involved in prostaglandin synthesis, and COX-2 plays a vital role in neovascularization by regulating vascular endothelial growth factor (VEGF) and fibroblast growth factor, migration of endothelial cells [30, 31]. Furthermore, Lactate dehydrogenase (LDH) is the primary metabolic enzyme that plays a significant role in regulating myocardial bioenergetics [6]. In the present study, treatment with Allicin significantly increased the myocardial LDH and COX-2 positive areas through immunofluorescence staining. Furthermore, western blot analysis demonstrated that p-AKT, p-PI3K, p-mTOR, COX-2, and VEGF protein levels were also increased in the Allicin group. These findings were in line with previous microarray analysis findings.

MicroRNAs (miRNAs) are members of the non-coding RNA family and have been reported to regulate gene expression through interactions with target genes [32, 33]. We have found that Allicin can promote angiogenesis by activating the PI3K-AKT-mTOR pathway, thereby improving myocardial I/R damage. Therefore, we hope to further explore the molecular mechanism of Allicin’s protective effect, and select miRNAs that have statistical differences and are related to angiogenesis for further research. In the current study, we performed miRNA microarray to examine the expressed miRNAs in MI/R tissues and corresponding normal tissues. A volcano map showed the differently expressed miRNAs between MI/R and normal controls. 67 genes were significantly downregulated, and 68 genes were upregulated. Gene Ontology (GO) annotations and Genomes (KEGG) pathway analysis found 35 upregulated pathways including regulation of angiogenesis and regulation of growth. Furthermore, the results of KEGG pathway analysis suggested that PI3K-AKT pathway was activated in the myocardial I/R group.

Among them, and we selected a miRNA for further investigation, miR-19a-3p, which served a critical role in cerebral ischemia/reperfusion [22]. In our previous experimental results, the number of neovascularization increased in the infarct area of the myocardial I/R group, and Allicin can further promote angiogenesis. At the same time, our experiments also detected the expression level of miR-19α-3p in the injured myocardial tissues and serums, and found that the expression level of miR-19α-3p was significantly down-regulated in the myocardial I/R group, while the expression of miR-19α-3p in the Allicin group was further reduced. This is consistent with the changing of angiogenesis. These results indicate that myocardial I/R can induce the expression of miR-19α-3p, which expression can be further regulated by Allicin. It indicates that miR-19α-3p may play an important role in the occurrence and development of myocardial I/R.

Evidence has indicated that LPS is a well-known M1 inducer that could produce proinflammatory cytokines in macrophages [25]. Tie2 expressing macrophages /monocytes (TEMs) play essential roles in the neovascularization of ischemic tissues [16]. *In vitro* study, LPS stimulated Tie2 expressing macrophages were cultured in an ischemic buffer. We found that the protein expression levels of p-PI3K, p-AKT, p-mTOR, VEGF, COX2, and MMP2 in LPS stimulated TEMs were gradually increased by Allicin treatment in comparison with the vehicle group. Furthermore, the accumulation of VEGF was noticeably visualized with fura-2/AM fluorescence in the Allicin treated TEMs. Next, we determined if PI3K pathway activation regulates VEGF and MMPs expression in TEMs. The pretreatment with Wortmannin, a specific PI3K inhibitor, reversed the increased p-PI3K, p-AKT, p-mTOR, VEGF, COX2, and MMP2 in TEMs.

As known, the PI3K signaling pathway has long been recognized as a dominant survival pathway regulating metabolism, growth, or survival [34]. Earlier work has indicated that miR-19a-3p activates PTEN and inhibits the PI3K/AKT pathway [35]. Here, we verify the regulation of miR-19a-3p on PI3K pathway by using neonatal mice tie2^+^ macrophages. MiR-19α-3p mimic / inhibitor were transfected to promote / inhibit the expression of miR-19a-3p, miRNA control and inhibitor control transfection being taken as control. And then, mice tie2^+^ macrophages were oxygenated for 4 hours and then reoxygenated for 2 hours to simulate the ischemia-reperfusion model *in vitro*. Compared with the control group, the expression of p-PI3K, p-AKT, p-mTOR, VEGF, Cox-2, MMP-2 were significantly decreased after miR-19α-3p mimic transfection, and further significantly increased after miR-19α-3p inhibitor transfection (Figure 6A and 6B). These data suggest that inhibition of miR-19a-3p promotes angiogenesis by regulating PI3K/AKT pathway.

In conclusion, the research of this study validated the protective effect of Allicin on myocardial I/R *in vivo* and *in vitro*. The present work revealed Allicin as a novel therapeutic target that attenuated myocardial ischemic injury through targeting the PI3K/AKT signaling pathway. We also revealed miR-19a-3p might be one of the crucial elements of the PI3K pathway in MI/R disease. Therefore, Allicin/ miR-19a-3p/ PI3K/AKT signaling pathway might denote the novel approach for MI/R prevention and treatment.
